# Infective endocarditis due to *Burkholderia cepacia* in a neonate: a case report

**DOI:** 10.1186/s13256-018-1633-z

**Published:** 2018-05-08

**Authors:** Emir Yonas, Vito Damay, Raymond Pranata, Nuvi Nusarintowati

**Affiliations:** 1 Faculty of Medicine, Yarsi University, Jakarta, Indonesia; 2 Faculty of Medicine, Pelita Harapan University, Tangerang, Indonesia; 3 Pediatric Cardiology Division, University of Indonesia, Jakarta, Indonesia

**Keywords:** Endocarditis, *Burkholderia*, Neonate

## Abstract

**Background:**

*Burkholderia* is a pathogen that is rarely seen in clinical cases. However, this organism is being found more commonly in hospitals.

**Case presentation:**

A female Indonesian newborn was referred to our neonatal intensive care unit because of respiratory distress. The newborn had been delivered the previous night via cesarean section. A physical examination revealed intercostal retractions and weak cry. The newborn’s gestational history was preterm, small for gestational age, and preterm premature ruptured membrane for 14 hours. Continuous positive airway pressure was administered. A multiple-antibiotic regimen consisting of ampicillin-sulbactam, gentamicin, meropenem, and ceftriaxone was initiated. Insertion of a central catheter was performed. The patient’s laboratory results were low blood albumin and globulin, anemia, and leukopenia. A blood culture revealed *Burkholderia cepacia* that was resistant to multiple antimicrobial agents. A chest x-ray showed infiltrate on both lung fields. Echocardiography showed two vegetations on the tricuspid valve.

**Conclusions:**

*B. cepacia* is a rare cause of infective endocarditis. With its capability to colonize water and grow on microbicides, the presence of *B. cepacia* in a patient’s blood warrants further investigation in institutions providing care. This might not be the first publication on this topic.

## Background

*Infective endocarditis* refers to infection of the valves of the heart. Nowadays, the cause of this disease has experienced a change in trend from various organisms usually found as the normal flora of the skin to organisms that have no place in any bodily surface as a normal flora. *Burkholderia* is one of these newly emerging pathogens causing infective endocarditis. Initially recognized as a cause of pulmonary infection in patients with cystic fibrosis in the 1980s, this microorganism has been found to cause endocarditis. This changing trend of the cause will introduce further challenges, difficulties, and, to some extent, threats into clinical practice worldwide. Advances made possible by technologies in medical science, such as the use of intravascular catheters in the management of patients in intensive care units, have been shown to be one of the many ways that these newly recognized organisms can find their way into the human body. *Burkholderia cepacia* is a microorganism originally intended to be used for agricultural purposes. The use of this organism began in the 1950s as a biopesticide. At that time, agricultural researchers found that this organism has the ability to antagonize a number of soilborne plant pathogens. These properties were later found to be mediated to some extent by antifungal substances secreted by the organism. *B. cepacia* emerged as a significant pathogen in patients with cystic fibrosis in the 1980s in a syndrome that consists of spiking fevers and severe progressive respiratory failure that proves to be devastating. The inability to treat such virulent infections was further compounded when the first patient-to-patient spread was reported in the early 1990s [1, 2].

In this report, we present a case of a right-sided infective endocarditis in a neonate with a positive culture for *B. cepacia*. We believe that the presentation of this case will benefit and contribute positively to medical science owing to the rare encounter of this organism as a pathogen in infective endocarditis and the difficulties in treating it.

## Case presentation

A female Indonesian newborn was referred to our neonatal intensive care unit because of respiratory distress. The newborn had been delivered the previous night via cesarean section. The newborn was delivered at a gestational age of 33 weeks (preterm delivery) by a G1P0A0 mother. The baby’s weight on delivery was 1200 g; her body length was 36 cm, and her Apgar scores at 1 and 5 minutes were 6 and 8, respectively. Prior to referral, the newborn had already received an intramuscular vitamin K injection at the referring facility. She was referred to our hospital aged 1 day old. Her physical examination findings were significant for intercostal retractions and a weak cry. The rest of the physical examination findings were within normal limits, which included a normocephalic head, bilateral icteric sclerae, bilateral nonanemic palpebral conjunctiva, normal eyes, no secretion from the ears or the nose, moist oral mucosa, no perioral cyanosis, normal S1and S2 heart sounds with no additional heart sounds, lung auscultation revealing vesicular breath sounds with no rales, a protuberant abdomen, liver able to be palpated with two fingers below the right costal arch, warm extremities, and capillary refill time less than 3 seconds. Her neurological examination revealed a lethargic newborn, but the rest of the neurological examination results were within normal limits, which included a normal head, normal sutures, normal fontanelles, no scalp swelling, and normal zone of transillumination. No abnormalities were detected by palpation of the spine. An inquiry on the gestational history revealed a preterm, small for gestational age fetus, with a transverse lie and premature rupture of the membranes for 14 hours. The mother denied any history of sickness during pregnancy. The patient’s parents denied any family history of illnesses of the heart and lungs. The patient’s family lives in a suburban home with adequate sanitation. The newborn was initially diagnosed with respiratory distress syndrome secondary to prematurity upon arrival to our facility. A continuous positive airway pressure device was administered to the patient, with a configuration of fraction of inspired oxygen 29%, positive end-expiratory pressure 6 cmH_2_O, and flow 8 cmH_2_O. A multiple-antibiotic regimen consisting of ampicillin-sulbactam and gentamicin was also administered. A peripherally inserted central catheter was placed.

The patient’s laboratory protein values upon admission revealed low blood albumin and globulin. Results of a blood panel obtained the day after admission were significant for anemia and leukopenia. A chest x-ray revealed suprahilar infiltrates on both of the lung fields and in the right pericardial region. No radiological anomalies of the heart were present. A repeat chest x-ray 2 days after admission revealed minimal suprahilar infiltrate on the right side, consistent with pneumonia. Meropenem and ceftriaxone were added to the antibiotic regimen on the fourth day of admission. Repeat blood panels obtained on days 5 and 8 after admission revealed severe anemia of 7.4 mg/dl, erythrocytopenia, leukopenia, and thrombocytopenia. Bilirubin panels showed increased elevated indirect bilirubin and low direct bilirubin, consistent with intrahepatic jaundice.

Tables 1, 2 and 3 present the results of the blood culture and resistance testing on the eighth day after admission. This was the only blood culture obtained in this patient.

**Table 1:** Results of blood culture and resistance testing

Type of examination: blood culture + resistance test Type of sample: blood Visual result: gram-negative rod Culture result: *Burkholderia cepacia*			
No.	Antibiotic	MIC (μg/dl)	Sensitivity
1	Ampicillin	> 32	R
2	Piperacillin-tazobactam	> 128	R
3	Cefepime	4	S
4	Ceftazidime	4	S
5	Meropenem	4	S
6	Amikacin	> 64	R
7	Gentamicin	> 16	R
8	Ciprofloxacin	2	R
9	Trimethoprim-sulfamethoxazole	< 20	S
10	Aztreonam	16	I
11	Tigecycline	4	I
12	Ampicillin-sulbactam	> 32	R
13	Cefazolin	> 64	R
14	Nitrofurantoin	> 512	R

*Abbreviations: S* Sensitive, *I* Intermediate, *R* Resistant, *MIC* Minimum inhibitory concentration

**Table 2:** Patient’s complete blood count and protein panel results on arrival

Examination type	Result	Reference range
Hemoglobin	15.7	14.5–22.5 g/dl
Hematocrit	43	45–67%
RBC	4.0	4.0–6.6 million/μl
WBC	7850	9000–30,000/μl
Platelets	199,000	150,000–400,000/μl
MCV	107	95–121 fl
MCH	39	31–37 pg
MCHC	37	29–37 g/dl
Blood type	O	
Rhesus	Positive	
Random blood glucose	106	< 140 mg/dl
CRP	< 6	< 6 mg/dl
Total Protein	3.8	6–8.5 g/dl
Albumin	2.4	3.5–5.0 g/dl
Globulin	1.4	2.5–3.5 g/dl

*Abbreviations: CRP* C-reactive protein, *MCH* Mean corpuscular hemoglobin, *MCHC* Mean corpuscular hemoglobin concentration, *MCV* Mean corpuscular volume, *RBC* Red blood cell count, *WBC* White blood cell count

**Table 3:** Patient’s blood gas analysis results on arrival

Examination type	Result	Reference range
pH	7.457	7.37–7.45
pCO_2_	26.5	33–44 mmHg
pO_2_	108.9	71–104 mmHg
HCO_3_^−^	18.9	22–29 mmol/L
Base excess	−3.1	−3 mmol/L
SO2	97.4	94–98%

*Abbreviations: HCO*_*3*_^*−*^ Bicarbonate, *pCO*_*2*_ Partial pressure of carbon dioxide, *pO*_*2*_ Partial pressure of oxygen, SO_2_ Oxygen saturation

Imipenem was added on the 12th day of admission. Protein panels showed reduced total protein, hypoalbuminemia, and hypoglobulinemia, with elevated prothrombin time, activated partial thromboplastin time, and red cell distribution width on the 14th day of admission. Repeat laboratory results on the 19th day of admission revealed microcytic hypochromic anemia, leukopenia, thrombocytopenia, and hypoalbuminemia.

**Fig. 1:**
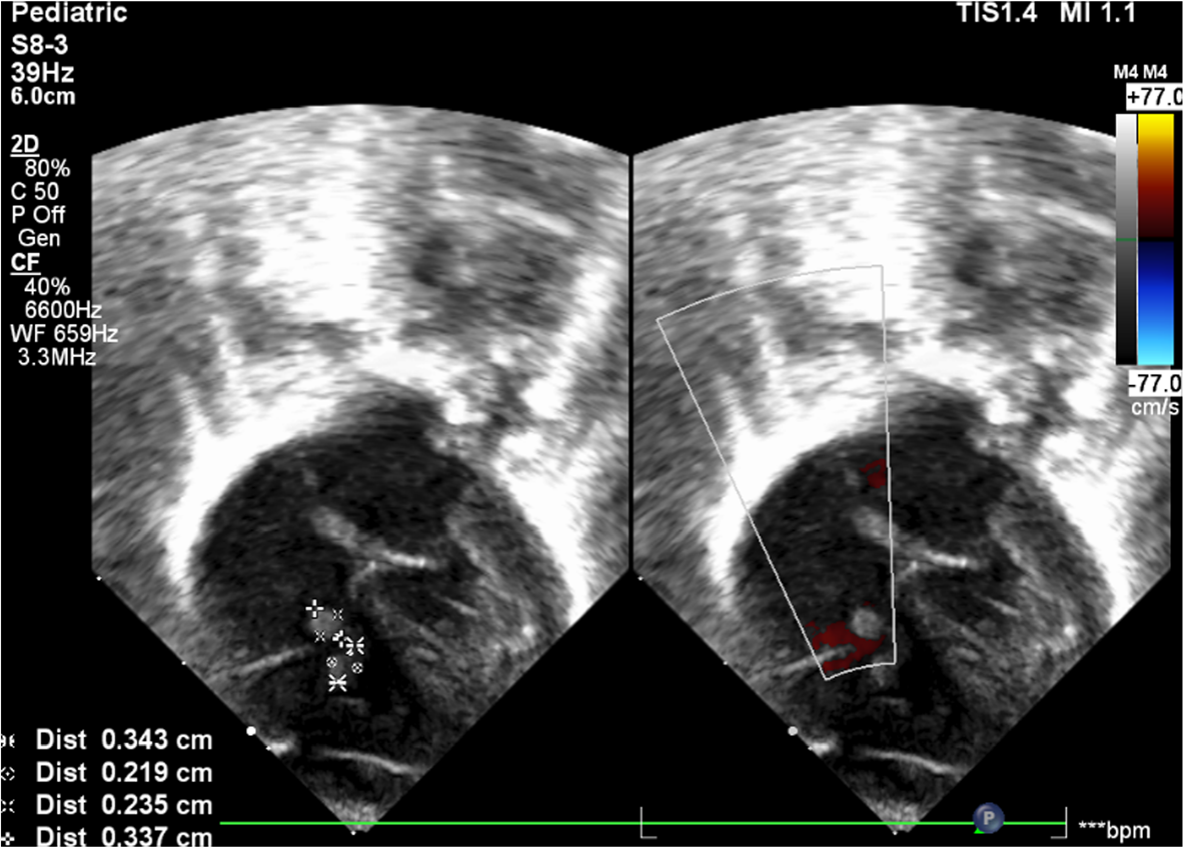
Echocardiography showing multiple vegetations

Echocardiography performed on the 23rd day of admission (Fig. 1) showed two vegetations on the tricuspid valve, with dimensions of 3.5 mm × 2 mm and 2.3 mm × 3.4 mm, respectively. On this basis, the diagnosis of infective endocarditis was established.

The following is a summary of medications that were administered to the patient prior to the diagnosis of infective endocarditis:

- Fat emulsion (SMOFLIPID™; Fresenius Kabi, Uppsala, Sweden) 20%, 0.3 cm^3^/hour
- Aminophylline 2 × 4 mg intravenous
- Imipenem 2 × 30 mg intravenous
- Metronidazole 2 × 10 mg intravenous
- Fluconazole 7 mg per 48 hours intravenous
- Acetaminophen 4 × 15 mg intravenous
- Furosemide 2 × 1 mg intravenous

The following is a timeline representing a summary of the patient’s clinical course at our facility:

- Day 1 postadmission: Edema of both lower extremities was observed; albumin 20% was administered
- Day 2 postadmission: Apnea reported with oxygen saturation (SO_2_) of 58% and heart rate of 70–80 beats per minute. Positive pressure ventilation was given, and postventilation SO_2_ was 90% with a heart rate of 134 beats per minute. Aminophylline was administered.
- Day 3 postadmission: Brownish fluid was observed in the orogastric tube. The patient was given nasopharyngeal oxygen. Transfusions of thrombocytes and fresh frozen plasma were given on the 3rd to 17th days of admission.
- Day 8 postadmission: Desaturation to 60% with a respiratory rate of 50 breaths per minute and heart rate of 180 beats per minute was reported. The patient was given an injection of dobutamine.
- Day 14 postadmission: The patient did not appear dyspneic. Noninvasive ventilation was omitted.
- Day 23 postadmission: Desaturation to 52% was reported. The patient was put back on noninvasive continuous positive airway pressure ventilation.
- Day 31 postadmission: The patient appeared dyspneic with intercostal retraction, with SO_2_ of 70%. Invasive ventilation using endotracheal intubation was administered.
- Day 32 postadmission:
  - 0600: Apnea was reported with SO_2_ of 45% on a ventilator. Positive pressure ventilation was performed.
  - 0645: The patient was declared dead. The patient had fallen into cardiac arrest; resuscitation efforts were unsuccessful; and mydriasis of both pupils was observed.

## Discussion

We report a case of a newborn with infective endocarditis caused by *B. cepacia* as determined by blood culture results. This case is unique in the literature owing to the rarity of this organism causing infective endocarditis. *B. cepacia* is an organism commonly found as a pathogen in pulmonary infections in patients with cystic fibrosis. The incidence of non-cystic fibrosis-related infection caused by this organism is low, which explains the unavailability of consensus guidelines on its treatment in patients without cystic fibrosis.

The estimated annual incidence of pediatric infective endocarditis in the United States ranges from 3.3 per 100,000 per year among infants younger than 1 year old to 0.3 to 0.8 per 100,000 per year in older children and adolescents [3, 4]. In a study using data from a pediatric health information systems database (2003 to 2010), the reported annual incidence of infective endocarditis ranged from 0.05 to 0.12 cases per 1000 pediatric hospital admissions [5]. Several risk factors for pediatric infective endocarditis have been identified, such as the presence of congenital heart disease, rheumatic heart disease, and central venous catheters. An indwelling central venous catheter, such as in the case of our patient, is a major risk factor for pediatric infective endocarditis [6]. Critically ill and premature infants are at risk. The immature immune systems of premature infants are thought to be one of the causes of susceptibility. The increased use of indwelling catheters in these pediatric groups appears to be a major factor in the development of infective endocarditis. The risk is also increased with the use of other intracardiac devices, such as ventriculoatrial shunts, pacemakers, prosthetic valves, and implantable cardioverter-defibrillators [7–11].

*Burkholderia* was once an agent used for agricultural purposes, but recently it has emerged as a threat to humans because it is becoming one of the most difficult infections to treat. The present case report shows that *Burkholderia* gains entry into the human body via the respiratory tract. This is supported by findings that respiratory macrophages, monocytes, and type II pneumocytes of the respiratory system, although all capable of performing phagocytosis, are unable to kill these bacteria. In these phagocytic cells, *Burkholderia* was found enclosed within membrane-bound vacuoles, which do not merge with the lysosomes in type II pneumocytes, or with a delayed fusion in macrophages. This report also reveals that a number of *Burkholderia* strains are well adapted to surviving and even replicating intracellularly in mammalian cells, especially those involved in innate immunity, such as the respiratory and epithelial phagocytic cells. All of these findings led the microbiologist to believe the Trojan horse hypothesis, in which amoeba living in aquatic environments, where they mostly feed on bacteria, contaminate drinking water. *Burkholderia* strains, however, with the capability of using amoeba by-products for their own purposes, are capable of surviving inside these amoebas. Contamination of drinking water by these amoebas will ultimately lead to infection in humans. Amoeba then will be trapped in the nasal mucosa and release small bacteria-containing vesicles that could infect the lower respiratory tracts [12].

*Burkholderia* has a tendency to colonize water pools and systems. This is possible because certain species of *Burkholderia* produce extracellular polysaccharides (EPSs). Several functions of this substance are attributed to its enhanced ability to survive in water, but not all of its functions are fully known. EPSs have protective functions by forming a physical barrier around cells and exhibiting more or less specific interactions with certain environmental molecules that are potentially dangerous for the bacteria, thus shielding the bacteria from this exposure. The unique property and structure of EPSs produced by *Burkholderia* may be used in a diagnostic approach for these bacteria [13].

One of the watery environments in which *Burkholderia* can survive is inside microbicides. This poses a huge dilemma and concern within medical practices because these microbicides are intended to kill bacteria in the first place. Antiseptics and disinfectants, as part of microbicidal substances, exhibit a broad spectrum of antimicrobial activity. An antiseptic is a germicide that destroys microorganisms, especially pathogenic ones. Alcohols, chlorhexidine gluconate, iodine, iodophors, quaternary ammonium compounds, and triclosan are commonly used as antiseptic agents. They rapidly penetrate into microorganisms and affect proteins, nucleotides, and fatty acids that are located in the bacterial structure and will ultimately result in cell death. Contamination in antiseptic substances may originate from the improper use of these products and can result in *Burkholderia* exposure to patients. There have been reports of outbreaks associated with chlorhexidine gluconate and benzalkonium diluted using water containing *Burkholderia*. Pharmaceutical water can also be a source of contamination with these bacteria in industrial settings [14]. Chlorhexidine is usually included in routinely used skin disinfection protocols. There have been reports of *Burkholderia* contamination in solutions of chlorhexidine diluted using water as opposed to solutions diluted using alcohol [15].

The molecular basis for biocide resistance in *Burkholderia* has been poorly studied, despite the organism’s being found in many instances contaminating disinfectants and other anti-infective solutions. Formulations containing benzalkonium chloride, cetylpyridinium chloride, and chlorhexidine have been found to be contaminated and have led to large outbreaks of infections. Even during manufacturing processes, disinfectants such as povidone-iodine may become contaminated with *Burkholderia*. Maintaining product sterility in manufacturing processes is a huge burden for pharmaceutical companies, and it has been a reason for several pharmaceutical recalls in the United States [16].

Difficulties in treating *Burkholderia* remain a major concern. Changes in reactive oxygen species are thought to be the major reason for difficulties in treating *Burkholderia*-caused infections. One study showed them not only to be antibiotic-dependent in production, in the sense that antimicrobial agents possess synergistic activities to increase production of reactive oxygen species, but also dependent on the time point at which starts to being produced. This is especially the case for biofilms because delay in production of reactive oxygen species in immunologic cells is seen when dealing with this unique structure. Biofilm, which has been known to be produced and generated by *Burkholderia*, can impede the recognition process by immune cells and thus further impede the production of reactive oxygen species against these bacteria [17].

This finding is further complicated by another finding that “persister” cells exist inside the biofilm produced by *Burkholderia*. These bacterial cells, which have a unique resistance to antimicrobial cells, exist exclusively within the biofilm. The Krebs cycle has been found to be downregulated in these cells, and the expression of genes involved in the electron transport chain was also downregulated. This is the mechanism by which these cells can avoid the production of reactive oxygen species [18].

*Burkholderia* was initially used to prevent molding of crops. However, studies have shown the antifungal agents produced by these bacteria to be pathogenic. These compounds, known as occidiofungins or burkholdines, have previously been shown to have antifungal activity, but they are now known to also possess high levels of hemolytic activity. These burkholdines have also been shown to be stable in the presence of human serum. This suggests that if burkholdines display pathogenic traits against humans, the compounds may resist degradation within the body. Furthermore, results of toxicological evaluation of burkholdines in mice did not show any organ toxicity, but they showed a reduction in body and organ weights. These findings suggest that the burkholdine-like peptides target and disrupt components of the membranes of eukaryotic cells (but not prokaryotic cells), especially erythrocytes, potentially binding to cholesterol or another cellular scaffolding carbohydrate component [19].

Secretion of tumor necrosis factor-α, interleukin (IL)-10, and IL-6 into the blood has been demonstrated to be significantly decreased in the presence of *B. cepacia* infection. It is presumed that the response of the host cells to *B. cepacia* secretory proteins mimics the physiological actions of the host toward toxins. This phenomenon may be referred to as *toxin tolerance*, which is due to reduced capacity of the cells to synthesize and/or to secrete proinflammatory cytokines and is hypothesized to be caused by a defect in transcriptional and or translational regulation of the host. Moreover, the susceptibility pattern of *Burkholderia* seems to be altered by the presence of certain cations *in vitro*, such as magnesium, potassium, and glucose. These findings, however, have not yet been incorporated into therapeutic guidelines [6, 20–22].

In pediatric patients, *Burkholderia* has been reported to cause sepsis and arthritis in patients without cystic fibrosis. In a report of a neonatal intensive care unit outbreak of *Burkholderia*, as in our patient, all of the patients were exposed to intravascular procedures such as central intravenous line insertion or, as in our patient, a peripherally inserted central catheter. Also, all of the patients were treated at an intensive care unit for some time. This signifies that the transmission of *Burkholderia* is mediated by water and that spread of this organism to equipment connected to intravascular devices is highly likely, posing concerns of spread into the patient’s bloodstream mediated by this colonized equipment. In all reports of *Burkholderia* infections, no occurrence of inflammation at intravascular catheter insertion sites has been described, whereas in all of the reports, these were infections that are deemed possible only if there had been seeding of the organism through these intravascular devices. In an investigation following a *Burkholderia* outbreak in a neonatal intensive care unit in Malaysia, it was found that the infection had spread from the hands of staff members to the patients, in whom the organisms then colonized the respiratory circuits and the intravenous lines. Because *Burkholderia* is a pathogen uncommonly found on human skin, this infection can only occur if the person involved was somehow exposed to water contaminated by *Burkholderia*. Implementation of infection control measures, such as fumigation, thorough handwashing techniques, screening of intensive care unit staff, disinfecting thermometers, and resorting shelf life, as well as a limitation of shelf life of distilled waters used from large containers to not more than 24 hours, was recommended by one author [23–27].

Our patient was initially treated with an empiric antibiotic regimen of ampicillin-sulbactam and gentamicin, which are both drugs commonly used in the treatment of infective endocarditis. The present authors understood that despite the recommendations regarding empiric regimens of vancomycin and gentamicin for the initial treatment of infective endocarditis, obtaining vancomycin was difficult, so ampicillin-sulbactam was used. Ampicillin-sulbactam has a great coverage of both gram-negative and gram-positive organisms, with ampicillin being an aminopenicillin agent. The pairing of ampicillin with sulbactam, a suicide inhibitor of penicillin-binding proteins, is commonly seen as a resistance mechanism to penicillin agents. Sulbactam will bind to these proteins, rendering them inactive, allowing ampicillin to function as a microbial agent. Gentamicin is an agent that has wide coverage for gram-negative organisms. However, the use of these agents in our patient did not produce significant improvement. As per the current guideline, a blood culture is performed with all patients with infective endocarditis prior to administration of antimicrobial agents to look for causal organisms and antimicrobial sensitivity. The result in our patient showed *B. cepacia* as a causal organism, as well as sensitivity to cefepime, ceftazidime, meropenem, and trimethoprim-sulfamethoxazole. Meropenem was chosen to be added to the antimicrobial regiment of our patient. The organism present in our patient showed extensive resistance to at least ten different antimicrobial agents tested. Our approach was in accordance with a recommendation by the Centers for Disease Control and Prevention (CDC) regarding the treatment of *B. cepacia*. The CDC states that this organism can be resistant to many common antibiotics and that decisions on the treatment of *B. cepacia* infections should be made on a case-by-case basis, referring to sensitivity testing results [28].

## Conclusions

*B. cepacia* is a rare pathogen in infective endocarditis. This organism gains entry into the human body through various intravascular devices. This organism is innately resistant to various antimicrobial agents, posing a serious threat. With the capabilities of this organism to colonize water and grow on microbicides, the presence of such an organism in a patient’s blood warrants a thorough investigation in facilities involved in the treatment of the patient because this organism may have already contaminated the water system, water containers, and the microbicides that are being used. The lessons learned from this case are that (1) *B. cepacia* is innately resistant to various microbicides, (2) this organism will transmit through water-based media, and (3) therapy for this organism must be tailored on the basis of the culture result.

### Patient’s parents’ perspective

The patient’s parents were stricken by the fact that their daughter suffered from a serious heart condition. Initially, the parents expressed disbelief and denial of the diagnosis. After explanations were given by the physicians, the parents expressed pessimism regarding their daughter’s prognosis. The parents understood that this is a condition that is hard to treat.

## Abbreviations

CDC: Centers for Disease Control and Prevention
CRP: C-reactive protein
EPS: Extracellular polysaccharide
HCO_3_^−^: Bicarbonate
I: Intermediate
IL: Interleukin
MCH: Mean corpuscular hemoglobin
MCHC: Mean corpuscular hemoglobin concentration
MCV: Mean corpuscular volume
MIC: Minimum inhibitory concentration
pCO_2_: Partial pressure of carbon dioxide
pO_2_: Partial pressure of oxygen
R: Resistant
RBC: Red blood cell count
S: Sensitive
SO_2_: Oxygen saturation
WBC: White blood cell count
